# Perioperative allogenic blood transfusion increases the incidence of postoperative deep vein thrombosis in total knee and hip arthroplasty

**DOI:** 10.1186/s13018-019-1270-2

**Published:** 2019-07-23

**Authors:** Tao Jiang, Kai Song, Yao Yao, Pin Pan, Qing Jiang

**Affiliations:** 1 State Key Laboratory of Pharmaceutical Biotechnology, Department of Sports Medicine and Adult Reconstructive Surgery, Nanjing Drum Tower Hospital, The Affiliated Hospital of Nanjing University Medical School, 321 Zhongshan Road, Nanjing, 210008 Jiangsu People’s Republic of China; 2 Laboratory for Bone and Joint Disease, Model Animal Research Center (MARC), Nanjing University, Nanjing, 210093 Jiangsu People’s Republic of China

**Keywords:** Allogenic blood transfusion, Deep vein thrombosis, Total knee arthroplasty, Total hip arthroplasty

## Abstract

**Background:**

Excessive blood loss caused by total joint arthroplasty (TJA) often increases the requirement for blood transfusion, which is associated with adverse outcomes. The purpose of this study was to determine the relationship between perioperative transfusion and postoperative DVT in TJA.

**Methods:**

This retrospective study reviewed medical records of 715 patients, who consecutively underwent primary unilateral total knee arthroplasty (TKA) or total hip arthroplasty (THA) at our institution between September 2015 and March 2017. Demographic, clinical and surgical parameters were introduced into the univariate analysis to find risk factors for DVT within postoperative 30 days. In order to identify if allogenic blood transfusion was independently associated with DVT, a multivariate logistic regression analysis was conducted to adjust for gender, age, body mass index (BMI), diagnosis, and type of surgery.

**Results:**

The incidence of perioperative allogenic blood transfusion was 12.4% (*n* = 89). Fifty-seven patients (8.0%) developed DVT after surgery. Univariate analysis demonstrated that there were differences between DVT group and non-DVT group in gender (*P* = 0.045), age (*P* < 0.001), BMI (*P* = 0.026), primary diagnosis (*P* = 0.001), type of surgery (*P* < 0.001), and transfusion rates (*P* = 0.040). After adjustment by using multivariate logistic regression analysis, transfusion appeared to be the independent risk factor for DVT in TJA (*P* = 0.001; OR = 3.9, 95%CI 1.8–8.4).

**Conclusion:**

We found that perioperative allogenic blood transfusion was significantly associated with DVT following TJA. In order to reduce the risk of DVT and other adverse outcomes, methods to decrease transfusion rates should be used in clinical practice.

## Introduction

Total joint arthroplasty (TJA) can cause substantial blood loss, which increases the risk of blood transfusion during the perioperative period. The reported transfusion rates are 11.0–19.6% in total knee arthroplasty (TKA) and 15.9–29.8% in total hip arthroplasty (THA), respectively [1, 2]. Patients receiving transfusion during TJA are at higher risk of surgical site infection, cardiopulmonary complications, prolonged length of hospitalization, increased costs, and even mortality [3, 4].

Transfusion has also proven to be associated with postoperative deep vein thrombosis (DVT) in fields outside of orthopedics, including general surgery [5], urology [6], cardiac surgery [7], vascular surgery [8], obstetrics, and gynecology [9, 10]. In patients with isolated orthopedic trauma, the transfusion of packed red blood cell is the risk factor for venous thromboembolism (VTE) [11]. Transfusion has also been reported to increase the risk of DVT in patients undergoing spine surgeries [12–15].

DVT represents a common complication after TJA and can lead to adverse clinical outcomes, especially life-threatening pulmonary embolism (PE). Numerous relevant risk factors have been identified, including older age, obesity, metabolic syndrome, cardiovascular disease, previous thrombosis history, and a high American Society of Anesthesiologists (ASA) grade [16–19]. However, the relationship between transfusion and DVT in TJA is rarely studied. Therefore, we conducted this retrospective study to determine the association between perioperative allogenic blood transfusion and DVT after TKA and THA.

## Methods

This retrospective study was approved by our institutional review board (IRB). We enrolled consecutive patients undergoing primary unilateral TKA or THA at our institution between September 2015 and March 2017. If a patient received staged bilateral arthroplasties during the research period, only the first procedure was included. We excluded patients with coagulation disorders or established DVT prior to surgery.

If patients were bedridden for more than 48 h due to femoral neck fracture, preoperative anticoagulants were used. For the other patients, we did not routinely perform thromboprophylaxis before surgery. If patients had been taking aspirin for cardiovascular diseases, they did not need to stop it before surgery. All surgeries were conducted under general anesthesia by 4 surgeons. TKA was performed using medial parapatellar arthrotomy. A pneumatic tourniquet was inflated before skin incision and deflated immediately after cementing the prosthesis. The modified Hardinge approach was employed in THA, and we routinely used a cementless prosthesis. Intravenous tranexamic acid (TXA) was used before tourniquet release in TKA and skin incision in THA.

We used rivaroxaban (10 mg once daily) or low-molecular-weight heparin (LMWH) (30 mg once daily) to prevent DVT after surgery. However, chemical thromboprophylaxis might not be considered if intraoperative excessive blood loss or great amount of postoperative drainage occurred. Additionally, intermittent pneumatic compression devices were applied, and patients were encouraged to do ankle pump exercises during the postoperative period. All patients’ both lower limbs were screened for DVT by Doppler ultrasound within 3–5 days after surgery. If patients visited outpatient clinic with DVT-related symptoms after discharge, evaluation of DVT with ultrasound was also performed. All events of symptomatic and asymptomatic DVT within postoperative 30 days were recorded. If a proximal DVT was diagnosed, a thrombolytic dose of rivaroxaban (10 mg twice daily) was applied. For patients diagnosed with distal DVT, the prolonged use of anticoagulants was adopted. Inferior vena cava filter was not used in any of the patients in this study.

During the operation, blood transfusion was performed if the level of hemoglobin (Hb) decreased lower than 9.0 g/dL. After the operation, patients received blood transfusion if their levels of Hb were less than 8.0 g/dL, or if they developed obvious anemic symptoms with a Hb level of less than 9.0 g/dL. None of the patients in this study received allogenic blood transfusion or underwent autologous blood predonation before surgery.

Patients’ demographic, clinical, and surgical information was collected. Patients were divided into DVT group and non-DVT group. We compared the differences between these two groups in gender, age, body mass index (BMI), coexisting illnesses, previous surgeries in ipsilateral lower extremity, preoperative medication, smoking history, primary diagnosis, type of surgery (TKA or THA), operation time, amount of intraoperative blood loss, postoperative anticoagulants, and transfusion rates. Qualitative variables were analyzed by a chi-square test and quantitative variables were analyzed by a *t* test. In order to determine if transfusion was independently associated with DVT, a multivariate logistic regression analysis was employed to adjust for gender, age, BMI, diagnosis, and type of surgery. All the statistical analysis was done using STATA version 12.0 (Stata Corp. LP, College Station, TX, USA).

## Results

A total of 715 patients were enrolled with a mean age of 65.1 ± 12.6 years (range 21–94 years). There were 208 males and 507 females. Three hundred and nineteen patients underwent TKA for osteoarthritis (OA) or rheumatoid arthritis (RA), and 396 patients underwent THA for OA, RA, femoral neck fracture, osteonecrosis of the femur head, developmental dysplasia of the hip, or ankylosing spondylitis affecting hips. Table 1 shows the characteristics of these patients.

**Table 1:** Demographic, clinical, and surgical characteristics of the patients

Characteristics	Total (*n* = 715)	TKA (*n* = 319)	THA (*n* = 396)
Gender			
Male (%)	208 (29.1)	58 (18.2)	150 (37.9)
Female (%)	507 (70.9)	261 (81.8)	246 (62.1)
Age, years (mean ± SD)	65.1 ± 12.6	67.5 ± 9.2	63.2 ± 14.5
BMI, kg/m^2^ (mean ± SD)	24.7 ± 4.3	26.1 ± 4.1	23.5 ± 4.0
Diabetes (%)	116 (16.2)	69 (21.6)	47 (11.9)
Hypertension (%)	322 (45.0)	178 (55.8)	144 (36.4)
Malignance (%)	30 (4.2)	13 (4.1)	17 (4.3)
Cardiovascular disease (%)	84 (11.7)	40 (12.5)	44 (11.1)
Stroke (%)	95 (13.3)	48 (15.0)	47 (11.9)
COPD (%)	11 (1.5)	5 (1.6)	6 (1.5)
Venous disease (%)	24 (3.4)	16 (5.0)	8 (2.0)
Previous surgery (%)	18 (2.5)	1 (0.3)	17 (4.3)
Preoperative aspirin (%)	99 (13.8)	52 (16.3)	47 (11.9)
Preoperative steroid (%)	27 (3.8)	7 (2.2)	20 (5.1)
Smoking (%)	91 (12.7)	23 (7.2)	68 (17.2)
Diagnosis			
OA (%)	333 (46.6)	292 (91.5)	41 (10.4)
RA (%)	36 (5.0)	27 (8.5)	9 (2.3)
Femoral neck fracture (%)	133 (18.6)	–	133 (33.6)
ONFH (%)	145 (20.3)	–	145 (36.6)
DDH (%)	60 (8.4)	–	60 (15.2)
AS (%)	8 (1.1)	–	8 (2.0)
Operation time, min (mean ± SD)	109.7 ± 29.0	119.4 ± 22.0	101.9 ± 31.4
Intraoperative blood loss, ml (mean ± SD)	258.8 ± 208.8	200.3 ± 136.3	306.0 ± 242.5
Chemical thromboprophylaxis			
None (%)	28 (3.9)	10 (3.1)	18 (4.5)
Rivaroxaban (%)	599 (83.8)	279 (87.5)	320 (80.8)
LMWH (%)	88 (12.3)	30 (9.4)	58 (14.6)

^a^*TKA* total knee arthroplasty, *THA* total hip arthroplasty, *SD* standard deviation, *BMI* body mass index, *COPD* chronic obstructive pulmonary disease, *OA* osteoarthritis, *RA* rheumatoid arthritis, *ONFH* osteonecrosis of the femur head, *DDH* developmental dysplasia of the hip, *AS* ankylosing spondylitis, *LMWH* low-molecular-weight heparin

Eighty-nine patients (12.4%) received allogenic blood transfusion during perioperative period. Among them, 15 patients were only transfused intraoperatively, 69 patients were only transfused postoperatively, and 5 patients were transfused both intraoperatively and postoperatively. Separately, the incidence of transfusion was 6.6% (*n* = 21) in TKA and 17.2% (*n* = 68) in THA, respectively.

DVT was developed in 8.0% of these patients (*n* = 57). There were 3 proximal DVTs and 54 distal DVTs. Six patients had thrombi in the intramuscular venous plexus of both lower limbs, the others developed thrombi only in the operated legs. Among the patients diagnosed with DVT, 15 presented with DVT-related symptoms, the others were diagnosed with asymptomatic DVT by regular ultrasound screening. The DVT rates in TKA and THA were 13.2% (*n* = 42) and 3.8% (*n* = 15), respectively.

According to univariate analysis for total patients, there were statistically significant differences in gender (*P* = 0.045), age (*P* < 0.001), BMI (*P* = 0.026), primary diagnosis (*P* = 0.001), type of surgery (*P* < 0.001), and transfusion rates (*P* = 0.040) between DVT group and non-DVT group (Table 2). The subsequent multivariate logistic regression analysis showed that transfusion was the independent risk factor for postoperative DVT (*P* = 0.001; OR = 3.9, 95% CI 1.8–8.4) after adjustment for gender, age, BMI, primary diagnosis, and type of surgery (Table 3).

**Table 2:** Univariate analysis of the risk factors for DVT in TJA

Variable	Non-DVT (*n* = 658)	DVT (*n* = 57)	*P* value
Female gender (%)	460 (69.9)	47 (82.5)	0.045^*^
Age, years (mean ± SD)	64.7 ± 12.8	70.6 ± 8.8	< 0.001^*^
BMI, kg/m^2^ (mean ± SD)	24.6 ± 4.2	25.9 ± 4.4	0.026^*^
Diabetes (%)	110 (16.7)	6 (10.5)	0.224
Hypertension (%)	291 (44.2)	31 (54.4)	0.139
Malignance (%)	29 (4.4)	1 (1.8)	0.338
Cardiovascular disease (%)	77 (11.7)	7 (12.3)	0.896
Stroke (%)	84 (12.8)	11 (19.3)	0.163
COPD (%)	10 (1.5)	1 (1.8)	0.890
Venous disease (%)	23 (3.5)	1 (1.8)	0.484
Previous surgery (%)	18 (2.7)	0 (0)	0.206
Preoperative aspirin (%)	92 (14.0)	7 (12.3)	0.721
Preoperative steroid (%)	27 (4.1)	0 (0)	0.119
Smoking (%)	86 (13.1)	5 (8.8)	0.350
Diagnosis	–	–	0.001^*^
TKA (%)	277 (42.1)	42 (73.7)	< 0.001^*^
Operation time, min (mean ± SD)	109.9 ± 29.3	107.7 ± 24.2	0.585
Intraoperative blood loss, ml (mean ± SD)	262.9 ± 213.5	211.8 ± 136.2	0.076
Chemical thromboprophylaxis	–	–	0.185
Transfusion (%)	77 (11.7)	12 (21.1)	0.040^*^

^*^P < 0.05 was considered statistically significant

^a^*DVT* deep vein thrombosis, *TJA* total joint arthroplasty, *SD* standard deviation, *BMI* body mass index, *COPD* chronic obstructive pulmonary disease, *TKA* total knee arthroplasty

**Table 3:** Multivariate logistic regression analysis to identify the independent risk factors for DVT in TJA

	OR	95%CI	*P* value
Female	1.395	0.669–2.911	0.375
Age	1.051	1.019–1.084	0.002
BMI	1.059	0.989–1.135	0.102
Diagnosis	–	–	0.888
TKA	4.221	0.492–36.226	0.189
Transfusion	3.887	1.789–8.446	0.001

^*^P < 0.05 was considered statistically significant

^a^*DVT* deep vein thrombosis, *TJA* total joint arthroplasty, *OR* odds ratio, *CI* confidence interval, *BMI* body mass index, *TKA* total knee arthroplasty

In TKA group, we did not find risk factors for DVT. The incidence of transfusion was numerically higher in DVT group than that in non-DVT group (11.9% vs. 5.8%), but this difference was not statistically significant (*P* = 0.136).

In THA group, gender (*P* = 0.011), age (*P* = 0.005), and transfusion (*P* = 0.002) were found to be associated with postoperative DVT in univariate analysis. After adjustment for gender, age, BMI, and diagnosis, transfusion appeared to be the independent risk factor for postoperative DVT (*P* = 0.011; OR = 4.3, 95% CI 1.4–12.9).

## Discussion

In this study, we retrospectively studied 718 patients undergoing primary unilateral TKA or THA to explore the relationship between perioperative allogenic blood transfusion and DVT within postoperative 30 days. We attempted to introduce as many potential confounders as possible into the analysis to clarify the correlation. Transfusion was found to be significantly associated with DVT following TJA. Patients receiving blood transfusion had a four times greater risk of postoperative DVT.

According to the limited existing evidence, the relationship between blood transfusion and postoperative DVT in TJA remains controversial. Gray et al. [20] conducted a study involving patients receiving elective THA, and revealed a higher incidence of blood transfusion in those with DVT. Additionally, Danninger et al. [21] analyzed the outcomes of blood transfusion in TKA and THA, and found transfusion increased the risk of postoperative DVT. However, Frisch et al. [22] did not find the relationship between transfusion and DVT in their patients undergoing TKA and THA. Therefore, more studies with larger sample size and longer follow-up time regarding this topic are required.

Blood transfusion can alter the local hemorheology and increase blood viscosity, which causes red blood cell (RBC) aggregation, and subsequent thrombus formation [23]. In addition, patients present with a hyper-inflammatory and hypercoagulable state after TJA. Transfusion stimulates neutrophils to release inflammatory cytokines such as IL-8 and sPLA2 [24]. The proinflammatory nature of allogenic blood transfusion may further increase the risk of thrombosis. It is also demonstrated that the transfusion of RBC can increase platelet aggregation [25], which may play a role in DVT formation. Besides, stored RBCs release free hemoglobin and microparticles, which decrease the level of nitric oxide and cause vasoconstriction [26]. Spinella et al. [27] performed a study to explore the effects of RBC storage duration on the incidence of DVT in patients with traumatic injuries, and found that the transfusion of RBC with a storage age of more than 28 days increased the risk of DVT significantly.

Apart from the risk of allergy and transmitted diseases, blood transfusion also increases the risk of adverse outcomes, longer length of stay, increased costs, and discharge to short-term care in patients undergoing TJA [28]. This study shows that transfusion is also the risk factor for postoperative DVT. Given the numerous latent risks of transfusion, it is critical to identify the patients at high risk of transfusion and to employ measures to reduce transfusion rates in TJA. Preoperative low level of Hb is strongly associated with postoperative transfusion [29–31]. Therefore, preoperative hemoglobin optimization has got widespread attention. Erythropoietin and iron supplementation have proven to be effective in decreasing the requirement of transfusion in TJA [32, 33]. Autologous blood predonation is also a useful preoperative strategy to reduce the need for allogenic transfusion [34, 35]. During the surgery, there are several methods to decrease blood loss, including the use of antifibrinolytic agents (TXA and aminocaproic acid), locally applied hemostatic agents (fibrin sealant and bone wax), and computer navigation [36–40]. In addition, intraoperative and postoperative blood salvages are used to decrease the need for and quantity of allogenic transfusion [41, 42]. However, blood salvage does not reduce allogenic transfusion rates in patients with a preoperative Hb level of less than 12 g/dL [43], so a combination of preoperative optimization should be considered in these patients.

It should be noted that this study has the limitations inherent to retrospective single-center study. There are still other confounders not introduced into the analysis due to the retrospective nature of this study. We excluded patients with coagulation disorders from this study. However, patients enrolled in this study may have other types of blood abnormality, such as anemia. Additionally, the small sample size did not allow us to perform subgroup analysis about the effects of doses or types of blood products on the incidence of DVT. There was a higher transfusion rate in THA group despite using TXA in this study compared to that in other studies, which may be caused by different blood management strategies. In this study, we only analyzed the DVTs occurring within postoperative 30 days. Those occurring outside of the period were not included in this study. There were only three patients diagnosed with proximal DVTs, and they did not receive blood transfusion during perioperative period. However, we cannot determine the relationship between blood transfusion and proximal DVTs due to such a small sample size.

## Conclusion

This study demonstrated the relationship between perioperative allogenic blood transfusion and postoperative DVT in patients undergoing primary unilateral TKA and THA. In order to reduce the risk of DVT and other adverse outcomes, strategies to decrease transfusion rates should be employed in clinical practice.

## Data Availability

The data used to support the findings of this study are available from the corresponding author upon request.

## Abbreviations

BMI: Body mass index
DVT: Deep vein thrombosis
IRB: Institutional review board
LMWH: Low-molecular-weight heparin
OA: Osteoarthritis
PE: Pulmonary embolism
RA: Rheumatoid arthritis
RBC: Red blood cell
THA: Total hip arthroplasty
TJA: Total joint arthroplasty
TKA: Total knee arthroplasty
TXA: Intravenous tranexamic acid
